# Unsupervised Tablet Defect Detection Method Based on Diffusion Model

**DOI:** 10.3390/s25175254

**Published:** 2025-08-23

**Authors:** Mengfan Zhang, Weifeng Liu, Linqing He, Di Wang

**Affiliations:** School of Electrical and Control Engineering, Shaanxi University of Science and Technology, Xi’an 710016, China; 230612052@sust.edu.cn (M.Z.); 230611024@sust.edu.cn (L.H.); 230611017@sust.edu.cn (D.W.)

**Keywords:** tablet anomaly detection, diffusion model, feature extraction, anomaly location, unsupervised detection method

## Abstract

Reconstruction-based unsupervised detection methods have demonstrated strong generalization capabilities in the field of tablet anomaly detection, but there are still problems such as poor reconstruction effect and inaccurate positioning of abnormal areas. To address these problems, this paper proposes an unsupervised **D**iffusion-based **T**ablet **D**efect **D**etection (**DTDD**) method. This method uses an Assisted Reconstruction (AR) network to introduce original image information to assist in the reconstruction of abnormal areas, thereby improving the reconstruction effect of the diffusion model. It also uses a Scale Fusion (SF) network and an improved anomaly measurement method to improve the accuracy of abnormal area positioning. Finally, the effectiveness of the algorithm is verified on the tablet dataset. The experimental results show that the algorithm in this paper is superior to the algorithms in the same field, effectively improving the detection accuracy and abnormal positioning accuracy, and performing well in the tablet defect detection task.

## 1. Introduction

Drug quality is directly related to the health and safety of patients and the brand reputation of pharmaceutical companies. In recent years, drug safety issues [1,2,3] have occurred frequently, and the number of drug safety accidents has continued to rise. Among them, drug surface defects [4,5] are an important component, a key quality issue that needs to be detected in the production process, and the most intuitive manifestation of product quality being affected. Therefore, drug surface defect detection is of great significance to ensure drug quality, improve drug safety, and reduce the frequency of accidents [6,7,8].

Tablets are the most common form of medicines. Tablet defect detection is generally aimed at detecting scratches, defects, foreign matter occlusion, color pollution, holes, and other defects on the surface of tablets [9]. Traditional manual visual inspection methods are inefficient and highly subjective, and it is difficult to meet the requirements of high-speed production lines for accuracy and real-time performance. Traditional image processing technology achieves abnormality recognition through low-dimensional feature extraction and rule design, but its generalization ability for complex texture defects is insufficient and it is easily affected by illumination changes and background interference. In recent years, deep learning technology [10,11,12,13] has significantly improved the accuracy and robustness of tablet defect detection with its powerful feature learning ability and has gradually become the mainstream method. Among them, supervised methods represented by Siamese Network [14], Fast R-CNN [15], and YOLO [16] have fully demonstrated their advantages of a high accuracy and good adaptability. TT Mac et al. [17] proposed a method that combines pill image processing with an improved VGG16 convolutional neural network based on Adam optimization to ensure a high accuracy of defect detection and classification. Xue Q et al. [18] applied YOLOV5 with attention mechanism and multi-scale feature fusion to the drug inspection scenario, providing a high-precision first-level real-time defect detection system. S Kim et al. [19] proposed a pipeline consisting of a pill detection module and a defect detection module based on an autoencoder to detect defective pills in pill packaging. For the task of tablet defect detection, supervised methods require a large amount of defect data annotation, which cannot cope with the problem of a small number of defect samples and diverse defect forms generated during the tablet production process. In this regard, unsupervised methods represented by DRAEM [20], PatchCore [21], and LDM [22] are more suitable, among which reconstruction-based methods are a major research hotspot [23]. The core of the reconstruction-based method is that the model only learns feature distribution from normal images during the training phase and uses the trained model to reconstruct abnormal images into normal images during the test phase so as to determine the abnormal location by comparing the reconstructed image with the input image. Autoencoders [24], Variational Autoencoders [25] and Generative Adversarial Networks [26] all belong to this category. In recent years, the diffusion model, which has performed outstandingly, has been widely studied and applied.

In 2015, Jascha Sohl-Dickstein et al. [27] proposed a diffusion model based on the principles of non-equilibrium thermodynamics. The idea is to systematically and slowly destroy the structure of the original data distribution through an iterative forward diffusion process so that it gradually becomes a simple, analyzable distribution, and then learn a reverse diffusion process to restore the structure in the data, thereby obtaining a highly flexible and easy-to-handle generation model. In 2020, Ho J et al. [28] proposed a model for image generation, and it has been widely used. In 2021, Robin Rombach et al. proposed the LDM architecture, which can greatly reduce the computational complexity and achieve better results by performing a diffusion process on the latent representation space. J. Wang et al. [29] proposed a defect detection model based on a classifier-guided conditional diffusion model to detect defects and accurately identify defective areas; Hang Yao et al. [30] proposed a global and local adaptive diffusion model that can achieve anomaly-free reconstruction while retaining normal information as much as possible. He H et al. [31] proposed a diffusion-based anomaly detection architecture for multi-class anomaly detection, which can cope with the anomaly detection problem under multiple classifications. Zhang X et al. [32] proposed a feature reconstruction network Realnet with real synthetic anomalies and adaptive feature selection to synthesize real and diverse anomaly samples. In addition, to address the limitations of feature extraction in anomaly detection tasks, Z He et al. [33] introduced a cross-modal recurrent enhancement module and a frequency-aware alternating fusion module. These modules effectively suppress interference from low-quality depth maps and facilitate the aggregation of cross-modal features. By overcoming the similarity between the foreground and background in the frequency domain, this approach enables robust cross-scale fusion of integrated features. S Woo et al. [34] proposed the CBAM attention mechanism, which infers attention maps along two independent dimensions, channel and space, and it is widely used to improve the representation ability of the network. J Chen et al. [35] proposed a bidirectional cross-scale connection feature fusion network with a direct information connection layer and a shallow information fusion layer to address the difficulty and low accuracy of small target detection. Although the above methods have achieved certain results, they still face many challenges in the task of tablet defect detection: (1) small target defects such as tiny scratches and pits on the surface of tablets have a very low pixel ratio and extremely weak semantic information. Existing detection algorithms find it difficult to accurately capture their features, resulting in large positioning deviations and difficulty in designing an adaptive loss function to optimize the detection effect. (2) The defect morphology of tablets varies greatly. It is difficult for the feature fusion network to balance the feature extraction at different scales. The shallow network cannot deeply explore the details of small defects, and the deep network is prone to losing the overall structural information of large defects, resulting in limited detection accuracy. (3) Some tablets have complex defect backgrounds and textures, resulting in poor reconstruction effect and reconstruction quality of existing algorithms. In this regard, this paper proposes an unsupervised tablet defect detection method, DTDD, based on a diffusion model.

The contributions of this paper are as follows:

(1) An AR network is used to improve the quality of image reconstruction. This network introduces the semantic information of the original image into the diffusion model denoising network, ensuring the consistency of semantic information and assisting the diffusion model to correctly reconstruct the abnormal image, thereby improving the reconstruction effect.

(2) A semantically enhanced lightweight attention module (SELA) is designed to enhance the semantic expression and detail perception of target features, thereby improving the ability of the AR network to understand global semantic information and local detail textures.

(3) An SF network is used to achieve accurate positioning of abnormal areas. This method achieves hierarchical extraction of features of different scales through explicit task division, performs abnormality measurement and adaptive weighted fusion on features of each layer, avoids the computational overhead of cross-layer fusion, and enables the model to take into account both details and global information.

## 2. Background

### 2.1. Denoising Diffusion Probabilistic Model

DDPM models the data generation process as a reverse Markov chain, which is divided into two stages: noise addition (forward process) and data generation (reverse process). In the forward diffusion process, the model starts from the data and gradually adds Gaussian noise until the signal is completely covered by the noise. Assume that $x_{0}$ is the original data, $q(x_{0})$ is the original data density, *t* represents the time of the diffusion step, $\beta_{t}$ is the preset variance schedule, which determines the amount of noise added, *I* represents the identity matrix of the same dimension as the input image $x_{0}$, ϵ is the noise sampled from the standard Gaussian distribution, Definition $\alpha_{t}:=1-\beta_{t}$, and ${\bar{\alpha }}_{t}:=\prod_{s=1}^{t}\alpha_{s}$, then the entire forward process $q(x_{t}|x_{0})$ can be expressed as follows:

(1) $$q\left(x_{t}|x_{0}\right)=N\left(x_{t};\sqrt{{\bar{\alpha }}_{t}}x_{0},\left(1-{\bar{\alpha }}_{t}\right)I\right)$$

(2) $$x_{t}=\sqrt{{\bar{\alpha }}_{t}}x_{0}+\sqrt{1-{\bar{\alpha }}_{t}}\epsilon$$

In the process of inverse denoising, the model learns to gradually recover the original noise-free data from the noisy data. Definition *p* is a parameterized Gaussian distribution, and its learning process can be expressed as follows:

(3) $$p_{\theta }\left(x_{t-1}\left|x_{t}\right|\right)=N\left(x_{t-1};\mu_{\theta }\left(x_{t},t\right),\Sigma_{\theta }\left(x_{t},t\right)\right)$$

(4) $$p_{\theta }\left(x_{0:T}\right)=p\left(x_{T}\right)\prod_{t=1}^{T}p_{\theta }\left(x_{t-1}|x_{t}\right)$$

$p_{\theta }\left(x_{t-1}|x_{t}\right)$ means the joint probability distribution of the current moment after the next time step data is given. The data distribution of the current time step is Gaussian distribution, and its mean and covariance are determined by the model parameters. $p_{\theta }\left(x_{0:T}\right)$ means the joint probability distribution from the last time step to the first time step. In the inverse denoising process, the model uses the maximum likelihood objective to train the neural network and uses stochastic gradient descent to optimize the random term. The expression can be written as follows:

(5) $$\begin{array}{cc} L & =D_{KL}\left(q\left(x_{T}|x_{0}\right)\|\;p\left(x_{T}\right)\right)\\ \; & +\sum_{t>1}D_{KL}\left(q\left(x_{t-1}|x_{t},x_{0}\right)\|\;p\left(x_{t-1}|x_{t}\right)\right)-logp\left(x_{0}|x_{1}\right) \end{array}$$

Set the covariance to a fixed value and calculate the mean as follows:

(6) $$\mu_{\theta }=\frac{1}{\sqrt{\alpha_{t}}}\cdot \left(x_{t}-\frac{1-\alpha_{t}}{\sqrt{1-{\hat{\beta }}_{t}}}\cdot z_{\theta }\left(x_{t},t\right)\right)$$

Among them, $z=\frac{x-\mu }{\sigma }$; this enables the model to more accurately reconstruct the original data from the noisy data, and the loss function is simplified to the following formula:

(7) $$L_{simple}\left(\theta \right)=E_{t,x_{0},\epsilon }\left[\epsilon -\epsilon_{\theta }{\left(\sqrt{{\bar{\alpha }}_{t}}x_{0}+\sqrt{1-{\bar{\alpha }}_{t}}\epsilon ,t\right)}^{2}\right]$$

### 2.2. Latent Diffusion Model

LDM first uses a pre-trained variational autoencoder to encode high-dimensional image data into a low-dimensional latent space. The representation in this latent space is more compact and computationally efficient than the original image space. The encoder maps image $x_{0}$ to latent space $z_{0}$, where LDM applies a diffusion model to generate data. The diffusion model transforms the data from the original distribution to a Gaussian distribution by gradually adding noise, and then it learns how to reverse the process. Finally, the decoder is used to reconstruct image ${\hat{x}}_{0}$ from the latent space. The expressions of the forward diffusion process and the reverse denoising process are shown in Equations (8) and (9).

(8) $$z_{t}=\sqrt{{\bar{\alpha }}_{t}}z_{0}+\sqrt{1-{\bar{\alpha }}_{t}}\epsilon_{t},\epsilon_{t}∼N\left(0,I\right)$$

(9) $$z_{t-1}=\frac{1}{\sqrt{\alpha_{t}}}\left(z_{t}-\sqrt{1-\alpha_{t}}\epsilon_{\theta }\left(z_{t},t\right)\right)$$

Since LDM supports conditional generation, the given conditional information *c* such as text description and category label can be introduced into the inverse diffusion process through the cross-attention mechanism to generate images. Therefore, its loss function can be expressed as follows:

(10) $$L=E_{z_{0},\epsilon ,t}\left[\|\epsilon -\epsilon_{\theta }\left(z_{t},t,c\right){\|}^{2}\right]$$

### 2.3. Tablet Surface Defect Types

**Figure 1:**
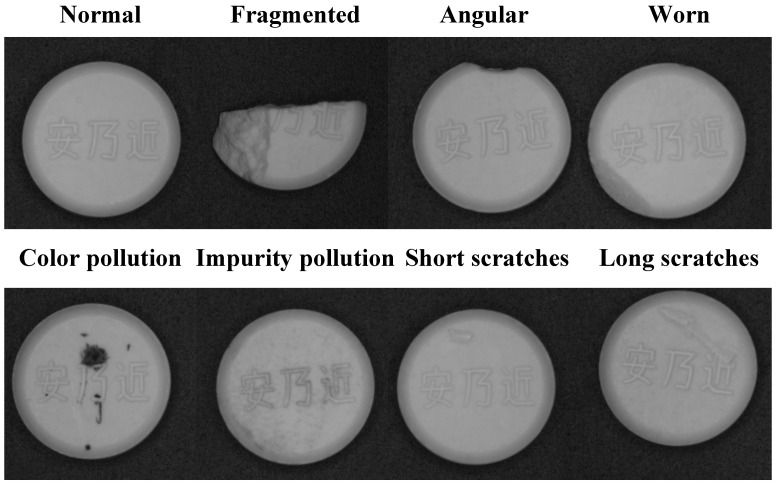
Tablet defect types.

The types of surface defects of tablets mainly include the following, as shown in Figure 1.

Tablet defect. The basic outline of the tablet is incomplete, and some structures are changed, including cracks, missing corners, wear, etc. The cracks are manifested as the tablet splitting from the top or bottom to form one or more fragments; the missing corners are mostly caused by external force collision, resulting in the missing corners of the tablet. Wear is due to local loss caused by friction and other reasons, resulting in an uneven tablet surface or shape change.

Tablet contamination. Discoloration, contamination, or impurities on the tablet surface, mainly including color contamination and impurity contamination.

Tablet surface scratches. This type of defect is generally caused by uneven molds or scratches by sharp objects. Depending on the depth and length of the scratches, the damage to the tablet structure is also different.

## 3. Method

The DDTD network structure proposed in this paper consists of a latent diffusion model guided by the AR network and an SF network for feature extraction. The LDM-AR network is used to generate high-quality reconstructed images, and the SF network is responsible for extracting multi-scale features and comparative analysis of the input image and the reconstructed image, and then outputting accurate anomaly scores. In terms of input images, we integrated tablet data from open source datasets such as the MVTec anomaly detection dataset (MVTec AD) [36] to construct a tablet defect detection dataset containing multi-scale defects and complex backgrounds. Combined with the field images collected by the self-built test bench, the model’s field detection performance was tested on it.

### 3.1. Network Architecture

In the image reconstruction task, in order to balance computational efficiency and image reconstruction quality, this paper introduces LDM as the core framework. The input image $x\in {ℜ}^{3\times H\times W}$ is first encoded into a latent space vector z=ε(x) through a pre-trained encoder, where $z\in {ℜ}^{c\times H\times W}$. Then, in the latent space, the vector is gradually noisy to become a random Gaussian noise vector $z_{t}$, completing the forward diffusion process of the diffusion model. The output $z_{t}$ of the forward diffusion process can be represented as follows:

(11) $$z_{t}=\sqrt{{\bar{\alpha }}_{t}}z_{0}+\sqrt{1-{\bar{\alpha }}_{t}}\epsilon_{t},\epsilon_{t}∼N\left(0,I\right).$$

In order to further enhance the model’s ability to understand the semantic information of the original image, this paper designs an AR network in the reverse denoising stage to guide the denoising network to reconstruct high-quality images. In this process, the random Gaussian noise vector $z_{t}$ is input into the denoising network of the diffusion model. At the same time, the AR network performs deep feature processing on the original image and the random Gaussian noise vector $z_{t}$ and fuses the calculation results with the denoising network output. Through this collaborative mechanism, the LDM is effectively guided to generate a reconstructed image $\hat{x}$ with richer details and more accurate semantics.

**Figure 2:**
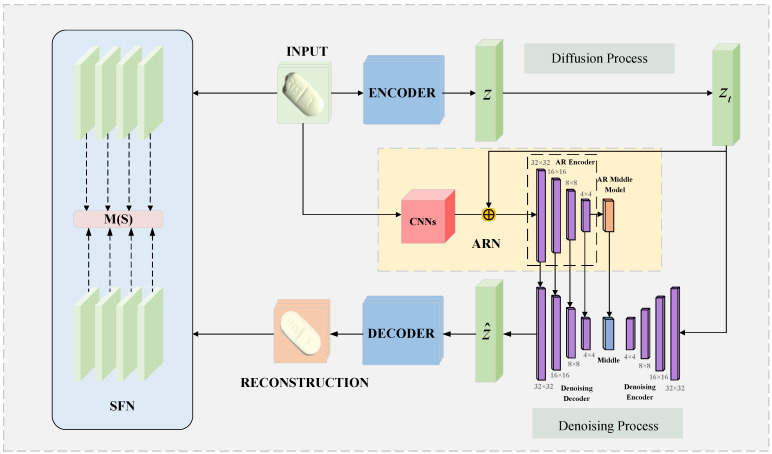
Model framework.

In the detection and location tasks of abnormal areas, we introduce the difference comparison between the two into the feature space. After obtaining the reconstructed image $\hat{x}$, it is input into the SF network together with the input image *x*. Through the multi-scale feature extraction strategy, the texture, shape, and structural differences of defects at multiple scales are effectively captured, thereby enhancing the recognition accuracy of various defects such as subtle scratches, tiny defects, and complex pollution. Taking into full consideration the characteristics of tablet defects, this paper adopts the combination of Euclidean distance and cosine similarity to design the anomaly score. By comparing the anomaly scores of input image *x* and reconstructed image $\hat{x}$ on feature maps of different scales, the abnormal area can be obtained. Finally, the abnormal area is visualized through heatmap generation to obtain the detection result. The DTDD network architecture is shown in Figure 2.

### 3.2. Assisted Reconstruction Network

**Figure 3:**
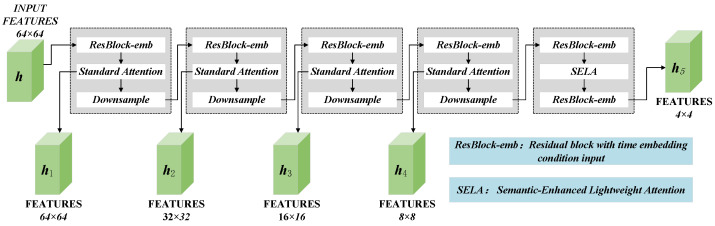
Implementation details of encoder in AR network.

In order to solve the problems of detail loss and semantic deviation in traditional diffusion models when reconstructing images, this paper designs an Assisted Reconstruction network. In the AR network, the input consists of two parts: input image *x* and random Gaussian noise vector $z_{t}$. The input image is first reduced to the same dimension as a random Gaussian noise vector through convolution. The sum of *x* and $z_{t}$ is then fed into the encoding block for continuous downsampling. Finally, the Assisted Reconstruction Middle (ARM) block extracts contextual information, gradually extracting and refining features and exploring deep connections between features, enabling the model to better understand the image content. The structure of the four encoding blocks and the ARM is shown in Figure 3.

The encoding block is mainly composed of a residual block with time-embedded conditional input, a standard attention mechanism, and a downsampling block. The current scale features extracted by each encoding block are saved and input into the downsampling block to achieve information transfer with the stable diffusion network. ARM mainly includes two residual layers and a semantically enhanced lightweight attention mechanism (SELA). The design of the double residual layer helps to extract deep features, and SELA can efficiently enhance the model’s ability to capture key semantic features.

The output of the ARM is superimposed with the middle block of the denoising network. The output of each encoding block is spliced with the output of the denoising network decoding block of the corresponding scale using jump connections, guiding the SD network from both global and local perspectives. In this way, the reconstructed output $\hat{z}$ covers both latent space features and original semantic features, which can fully focus on multi-scale information and achieve an accurate reconstruction of the tablet image.

After the denoising process is completed, the reconstructed output $\hat{z}$ is restored to the reconstructed image $\hat{x}=D\left(\hat{z}\right)$ through the pre-trained decoder, and the loss function can be expressed as follows:

(12) $$L_{DTDD}=E_{z,c_{ARN},\epsilon ∼N\left(0,1\right),t}\left[{∥\epsilon -\epsilon_{\theta }\left(z_{t},t,c_{ARN}\right)∥}_{2}^{2}\right]$$

where $c_{ARN}$ represents the auxiliary reconstruction information generated by the AR network.

### 3.3. Semantic-Enhanced Lightweight Attention

**Figure 4:**
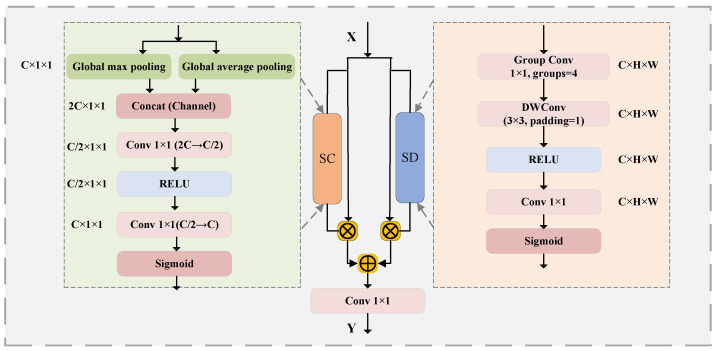
Schematic diagram of SELA.

In order to make the AR network pay attention to global semantic information and accurately capture local detail textures, this paper designs a semantically enhanced lightweight attention (SELA) module to enhance the semantic expression and detail perception capabilities of the target features. The module structure and process are shown in Figure 4. The SELA attention module adopts a parallel dual-branch structure. The semantic channel branch (left) and the spatial detail branch (right) enhance the feature expression capabilities from different dimensions and finally fuse to generate a semantically enhanced feature map. The semantic channel branch generates channel-level statistical features through average pooling and maximum pooling operations in the spatial dimension, and then generates SC attention weights through lightweight convolution to focus on channels with semantic significance. The spatial detail branch mines intra-channel associations through grouped convolutions and uses depthwise separable convolutions to enhance spatial detail features, generating SD attention weights to highlight the spatial area where the semantic information is located. The weights generated by the two branches weight the original feature maps respectively, and finally the enhanced results of the two branches are fused through splicing and 1 × 1 convolution.

Define the input feature map as $X\in R^{C\times H\times W}$ and the global maximum pooling and global average pooling results as $X_{max}=GlobalMaxPool\left(X\right)\in R^{C\times 1\times 1}$ and $X_{a\nu g}=GlobalAvgPool\left(X\right)\in R^{C\times 1\times 1}$; then, the SC attention weight generated by the semantic channel branch is expressed as follows:

(13) $$A_{SC}=Sigmoid\left\{{Conv}_{1}\left[RELU\left({Conv}_{1}\left(Concat\left(X_{\max},X_{a\nu g}\right),\frac{C}{r}\right)\right),C\right]\right\}$$

Among them, Sigmoid(·) is the Sigmoid function, RELU(·) is the RELU activation function, ${Conv}_{1}\left(\cdot ,k\right)$ is the 1 × 1 convolution operation, and the number of output channels is *k*; Concat(·) is the pooling splicing operation, and *r* is the channel compression ratio, which is set to 4 in this paper.

Define the group convolution of *X* to be divided into *G* groups, $X_{group}=GroupConv_{1}\left(X,G\right)$, and the depth-wise separable convolution operation DWConv can be defined as $X_{dw}=DWConv_{3}\left(X_{group},padding=1\right)$. Then, the SD attention weight calculated by the spatial detail branch is expressed as follows:

(14) $$A_{SD}=Sigmoid\left({Conv}_{1}\left(X_{dw},C\right)\right)$$

The output $Y\in R^{C\times H\times W}$ of the SELA module is expressed as follows:

(15) $$Y={Conv}_{1}\left(Concat\left(X⊙A_{SC},X⊙A_{SD}\right),C\right)$$

### 3.4. Scale Fusion Network

We feed the reconstructed image $\hat{x}$ and the input image *x* into the Scale Fusion (SF) network for feature extraction and calculate the anomaly score and locate the abnormal area based on the feature differences between the two types of images.

**Figure 5:**
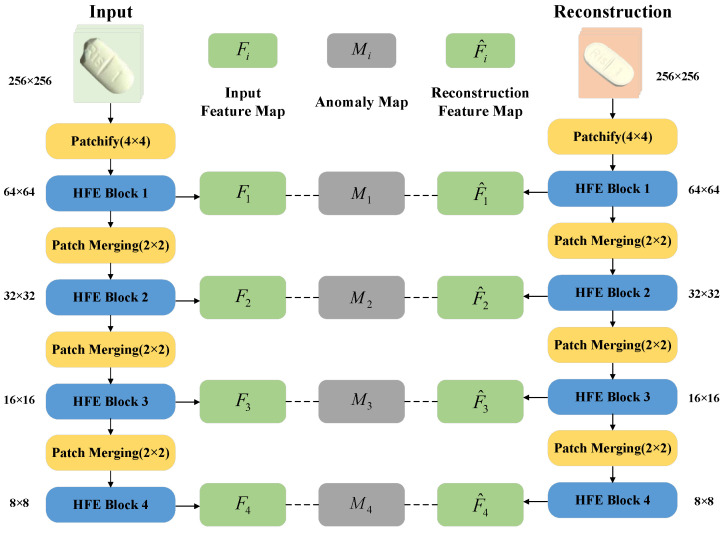
Schematic diagram of SF network.

The SF network proposed in this paper designs four hierarchical feature extraction blocks (HFE Block) for the task of tablet anomaly detection. It realizes the hierarchical extraction of features of different scales through explicit task division, avoiding the computational overhead of cross-layer fusion. The network framework is shown in Figure 5.

Among them, after sampling the Patchify (4 × 4) table, the initial features are extracted by 7 × 7 deep separable convolution to retain the macro texture of the tablet surface; HFE Block2 enhances the sensitivity to subtle defects by downsampling through Patch Merging and stacking 3 times of 3 × 3 deep separable convolutions; HFE Block3 introduces dilated convolution to expand the receptive field and adapt to defects of different sizes; HFE Block4 realizes global semantic modeling of the overall shape of the tablet through downsampling, 7 × 7 deep separable convolution, and global average pooling. Taking the input image X as an example, the calculation process of its multi-scale feature map $F_{i}$ is as follows:

(16) $$\begin{array}{cc} F_{1} & =PWConv(GELU\left(LN\left(DWConv\left(Conv_{4}\left(X\right);K_{7\times 7}\right)\right)\right))\\ \; & +Conv_{4}\left(X\right) \end{array}$$

(17) $$\begin{array}{cc} F_{2} & =PWConv(GELU\left(LN\left(DWCom^{3}\left(Down_{2}\left(F_{1}\right);K_{3\times 3}\right)\right)\right))\\ \; & +Down_{2}\left(F_{1}\right) \end{array}$$

(18) $$\begin{array}{cc} F_{3} & =PWConv(GELU\left(LN\left(DilConv\left(Down_{2}\left(F_{2}\right);K_{3\times 3},d=2\right)\right)\right))\\ \; & +Down_{2}\left(F_{2}\right) \end{array}$$

(19) $$\begin{array}{cc} F_{4} & =GAP(PWConv\left(GELU\left(LN\left(DWConv\left(Down_{2}\left(F_{3}\right);K_{7\times 7}\right)\right)\right)\right)\\ \; & +Down_{2}\left(F_{3}\right)) \end{array}$$

**Figure 6:**
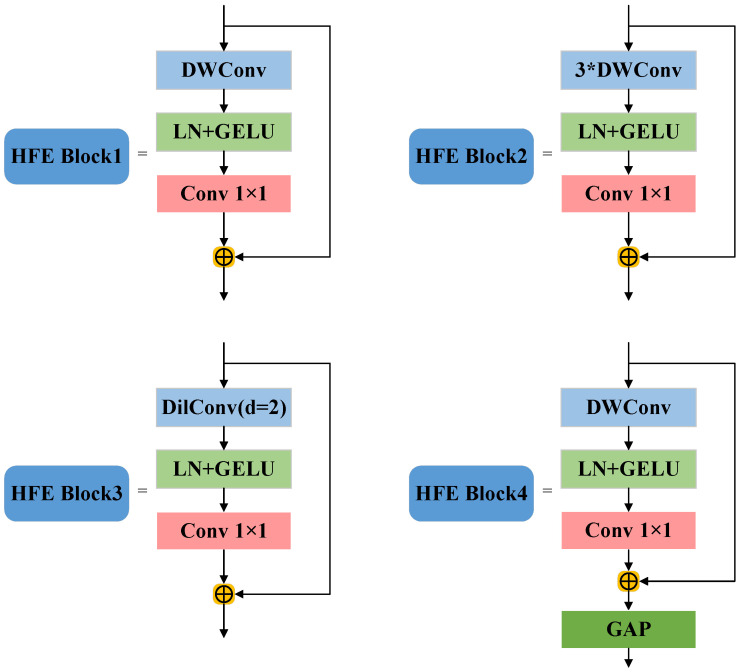
Schematic diagram of HFE blocks.

Among them, $Conv_{4}\left(\cdot \right)$ is a 4×4 convolution, $DWConv^{3}$ is a three-dimensional separable convolution stack, DilConv is an expansion convolution (expansion rate *d* = 2), $Down_{2}$ is a 2 × 2 downsampling operation, LN is layer normalization, GELU is a Gaussian error linear unit activation function, PWConv is a 1 × 1 pointwise convolution, and GAP is global average pooling. The structural diagram of the four blocks is shown in Figure 6.

The feature maps output at each stage have different resolutions and semantic information. We measure the abnormal distance between the input image feature map $F_{i}$ and the reconstructed image feature map ${\hat{F}}_{i}$ of the corresponding scale, and we obtain the abnormal feature map $M_{i}$ at different scales. Through the adaptive weighted fusion strategy, we use the learnable weights to upsample and fuse the feature maps of different scales, so that the model takes into account both details and global information. Compared with traditional models, the SF network can capture tiny defects and handle overall shape anomalies in the task of tablet anomaly detection, achieving a balance between accuracy and speed.

### 3.5. Anomaly Scores and Visualization

In the design of anomaly scores, cosine similarity, Mahalanobis distance, and Euclidean norm are the most commonly used calculation methods. Among them, Euclidean distance can measure the straight-line distance between two points in space, which is suitable for capturing the geometric shape, size, and other features of tablet images, thereby measuring such differences. Cosine similarity can measure the directional similarity of two vectors, which is suitable for capturing the texture, color distribution, and other features of tablets. Both are aimed at defect features in different directions and are simpler to calculate than Mahalanobis distance, which can improve the calculation speed. Considering the complex surface texture of tablets, many types of defects, and small individuals, we adopt a weighted combination of cosine similarity and Euclidean distance to construct anomaly scores. We calculate the anomaly scores of the input image features $F_{i}$ and reconstructed image features ${\hat{F}}_{i}$ of different scales extracted by the SF network, and the expression of the Euclidean distance $d_{E_{i}}$ of the i-th scale is

(20) $$d_{E_{i}}=\sqrt{\sum_{j=1}^{n}{\left(F_{ij}-{\hat{F}}_{ij}\right)}^{2}}$$

In order to achieve the weighting of cosine similarity and Euclidean distance, cosine similarity $s_{C}$ should be converted into cosine distance $d_{C}$, and the formula of the cosine distance $d_{C_{i}}$ of the *i*-th scale is as follows:

(21) $$d_{C_{i}}=1-s_{C_{i}}=1-\frac{F_{i}\cdot \hat{F_{i}}}{|F_{i}\|\hat{F_{i}}|}$$

Then, the expressions of the abnormal feature map $M_{i}$ and abnormal score *S* of the *i*-th scale are as follows:

(22) $$M_{i}=\lambda d_{E_{i}}+\left(1-\lambda \right)d_{C_{i}}$$

(23) $$S=\sum_{i=1}^{n}\omega_{i}Upsample\left(M_{i},\gamma_{i}\right)$$

Among them, Upsample(·) is the upsampling operation, $\gamma_{i}$ is the upsampling factor of the *i*-th scale, *n* is the number of feature maps involved in the fusion, and λ and $\omega_{i}$ are the weight coefficients of the distance metric and the multi-scale feature map, respectively. In order to display the calculated anomaly score results more concisely and clearly, and visually locate the defect area at the same time, we first use the anomaly score to generate anomaly maps and perform normalization and color mapping to obtain a more understandable visualization heat map. Similar operations are performed on the input image, and the heat map and the original image are superimposed, with the weights set to 0.6 and 0.4. The generated visualization results can display the abnormal area while retaining the original image information.

## 4. Experiment

### 4.1. Experimental Dataset

This study used multiple open-source datasets, including MVTec (https://www.mvtec.com/company/research/datasets/mvtec-ad, accessed on 19 August 2025) and PILL (https://aistudio.baidu.com/datasetdetail/121965, accessed on 19 August 2025), containing 3623 images of normal tablets and 1344 images of tablets with various defects, such as scratches, defects, stains, mold, bumps, and adhesions, mimicking actual industrial production scenarios. Of the 4967 tablet images in the dataset, the training set consists of 3586 normal tablet images, and the test set consists of 1381 images, including both normal and abnormal samples.

### 4.2. Implementation Details

We conducted experiments on a Linux deep learning server using the Ubuntu 20.04 operating system; the CPU model was 14 vCPU Intel (R) Xeon (R) Platinum 8362 CPU@2.80GHz, the GPU model was RTX3090, the video memory size was 48 GB, and the memory size was 64 GB. All models used were implemented based on Pytorch 3.9 using the PYtorch 1.12.1 framework, the Cuda version was 11.3, the image size was uniformly set to 256 × 256, the training rounds were 500 times, the batch size was 16, and all parameters were kept consistent in the experiments. All experimental results in this paper are presented as the mean ± standard deviation of five independent runs, and the significance of the improvements was verified by a *t*-test.

### 4.3. Evaluation Metrics

The evaluation indicators of anomaly detection usually include recall rate, precision, F1 score, AUROC, average precision, and the per-region-overlap score of each region, which are used to evaluate detection efficiency and accuracy. Unsupervised anomaly detection is characterized by a scarcity of anomaly samples, a high proportion of normal samples, class imbalance, and ambiguous anomaly definitions. The task requires both classification and localization. Therefore, evaluation metrics must meet the following requirements: be robust to imbalanced data, measure discriminative ability, and support region-level evaluation.

A single metric alone cannot meet these requirements. Therefore, this paper uses a combination of AUROC, PRO, F1, and AP as its evaluation metric. AUROC is insensitive to imbalanced data, eliminates the need for manual thresholding, and measures overall discriminative ability. AP is a comprehensive metric that combines ranking and localization but is sensitive to the ratio of positive and negative samples. F1 balances precision and recall, assessing overall performance at a practical threshold. PRO is a key metric for region-level anomaly localization, complementing the previous three metrics that ignore “regional details”. The formula is shown as follows:

The AUROC evaluation model is used as Formula (24) to evaluate the overall ability of the model to distinguish normal and abnormal samples under different thresholds. The closer the value is to 1, the better the model distinguishes between positive and negative samples.

(24) $$AUROC=\int_{0}^{1}\left(TPR\right)d\left(FPR\right)$$

The PRO index shown in Formula (25) is used to evaluate the overlap between the abnormal area predicted by the model and the actual abnormal area, and to measure the accuracy of locating the abnormal position.

(25) $$PRO=\frac{1}{N}\sum_{n=1}^{N}\frac{P\cap G_{n}}{G_{n}}=\frac{1}{N}\sum_{n=1}^{N}\frac{TP_{n}}{TP_{n}+FN_{n}}$$

The F1 score shown in Formula (26) is used to comprehensively measure the accuracy and recall of the model in detecting anomalies and to more comprehensively determine the accuracy of the algorithm.

(26) $$F_{1}=2\times \frac{Precision\times Recall}{Precision+Recall}$$

AP is used to judge the detection accuracy of the model for abnormal samples. By calculating the average precision under different recall rates, the accuracy of the model in detecting anomalies is reflected. Its expression is shown in Formula (27).

(27) $$AP=\int_{0}^{1}p\left(r\right)d\left(r\right)$$

### 4.4. Experimental Results

#### 4.4.1. Comparison with Baseline Model

The improvement of the algorithmic framework in the image reconstruction part is a targeted innovation based on the LDM algorithm process and the requirements of the tablet defect detection task. Therefore, this paper first compares the improved algorithm with the LDM algorithm to verify the advanced nature of the AR network. The experimental results are shown in Table 1.

It can be seen from the table data that the algorithm proposed in this paper performs excellently across all metrics. After verification by the *t*-test method, the results show that, compared with the LDM model, each metric achieves a significant improvement. The AP index increases by 11.15%, the F1 score increases by 9.61%, the AUROC increases by 14.61%, and the PRO increases by 15.73%. This shows that the AR network plays an important role in improving the overall accuracy of the model and guiding the model to perform image reconstruction.

#### 4.4.2. Comparative Experiment

**Figure 7:**
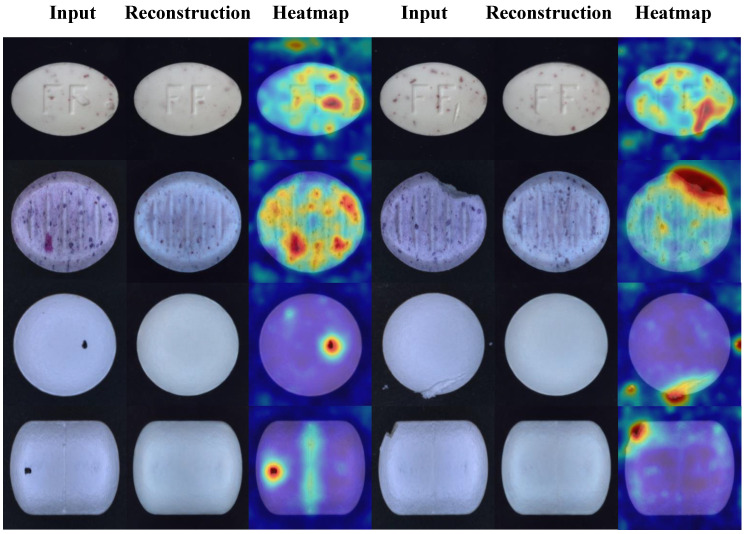
Visualization result.

The improved algorithm is improved and designed for the task of tablet defect detection and can cope with various defect types and small sample challenges. The visualization results of the test are shown in Figure 7. To further verify the superiority of the algorithm in the task of tablet defect detection, the algorithm in this paper is compared with five algorithms: UniAD [37], PaDiM [38], RD4AD [39], DRAEM, and YOLOV8. The experimental results are shown in Table 2.

**Figure 8:**
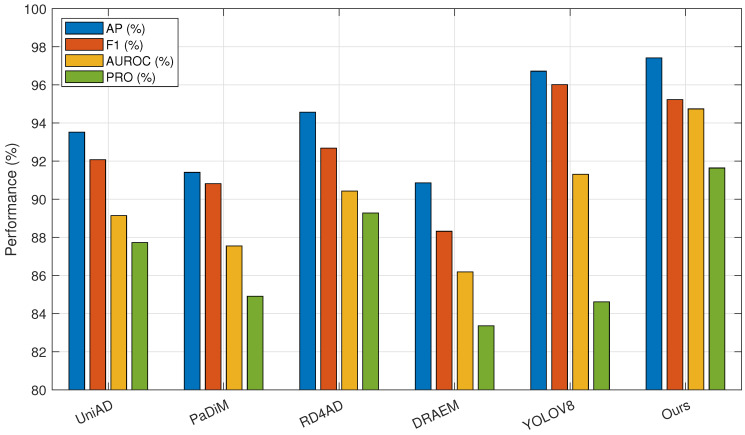
Comparative experimental results bar graph.

**Figure 9:**
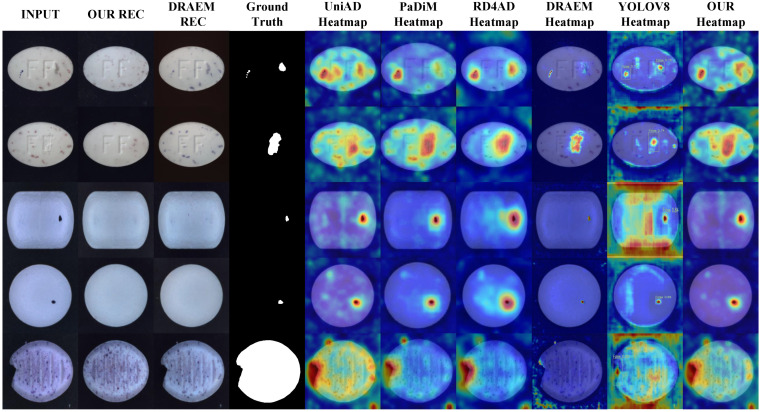
Qualitative illustration on Tablet dataset.

As shown in the table, the proposed algorithm achieves the best detection performance. Compared with the supervised algorithm YOLOV8, our method yields comparable results in AP and F1 metrics but demonstrates significant improvements in AUROC and PRO. Compared to the best results among other unsupervised algorithms, AP, F1, AUROC, and PRO are improved by 2.85%, 2.55%, 4.31%, and 2.36%, respectively. Therefore, our algorithm outperforms UniAD, PaDiM, RD4AD, DRAEM, and YOLOV8 in tablet defect detection tasks. To provide a more intuitive comparison between models, the results are presented in the form of bar charts in Figure 8. Figure 9 illustrates the performance of each algorithm on different tablet detection tasks within the dataset. As shown in the figure, the proposed algorithm exhibits superior reconstruction performance and sensitivity to anomalous regions, enabling precise detection of small defects of various types and shapes.

#### 4.4.3. Ablation Experiment

In addition to the AR network, this paper mainly makes innovative improvements in the three aspects of attention mechanism, feature extraction network, and anomaly score measurement, so ablation experiments were conducted on these three aspects to prove the effectiveness of the improvements.

The improvement of this paper in the attention mechanism is that it innovatively proposes the SELA attention mechanism to efficiently enhance the model’s ability to capture key semantic features. It is now compared with the three commonly used attention mechanisms of Self-Attention [40], CBAM, and ECA-Net [41]. The four attentions were used to train the model under the same parameters and to test the indicators. The data in Table 3 are as follows:

From the data in Table 3, it can be seen that the SELA attention proposed in this paper has better performance. Compared with other attentions, it has an improvement of 2.14%, 3.96%, and 2.01% in AP, AUROC, and PRO, respectively. Among them, SELA is slightly worse than ECA in F1 score, which is related to the strong morphological texture feature capture ability of the ECA local cross-channel interaction structure. In comparison, SELA has a certain gap, but it has more advantages in distinguishing boundary samples and multi-class defects.

**Figure 10:**
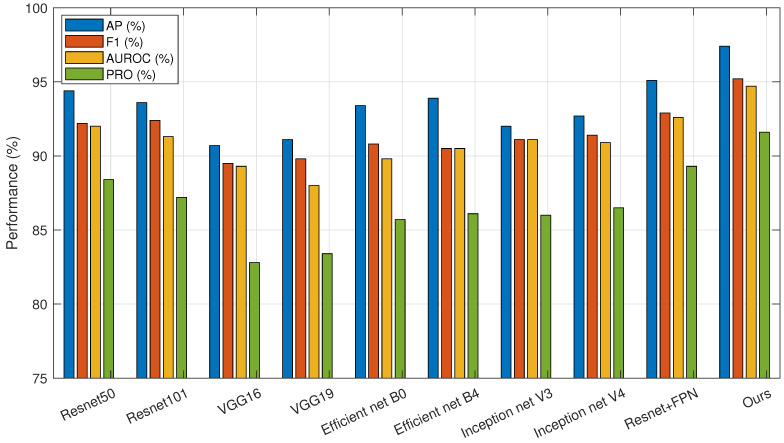
Ablation experiment results bar graph.

The improvement made in this paper in the feature extraction part is that it adopts an SF network with stronger feature extraction ability and multi-scale target processing ability. In order to verify the effectiveness of this network, different feature extraction networks were combined with the model of this paper, and experiments were carried out separately. The experiment replaced the SF network with representative models of ResNet, VGG, Efficient net, and Inception net series, and it added the combined structural comparison of ResNet50+FPN to increase the persuasiveness of the experiment. The ablation experiment results are shown in Table 4, which are organized into a bar chart format as shown in Figure 10.

From the data in the table, we can see that although the combination of ResNet+FPN has a certain improvement compared with other networks, its computational complexity is larger, which will cause a large waste of computing power. The algorithm in this paper not only has a lower computational complexity than the combination of ResNet+FPN, but also outperforms other feature extraction networks in various indicators and has better results in tablet defect detection. Among the four indicators, AP is 2.28% higher than the best data in other algorithms, F1 is 2.34% higher than the best data in other algorithms, AUROC is 2.10% higher than the best data in other algorithms, and PRO is 2.33% higher than the best data in other algorithms, which further proves the advantages of the algorithm in this paper in the task of tablet defect detection.

**Figure 11:**
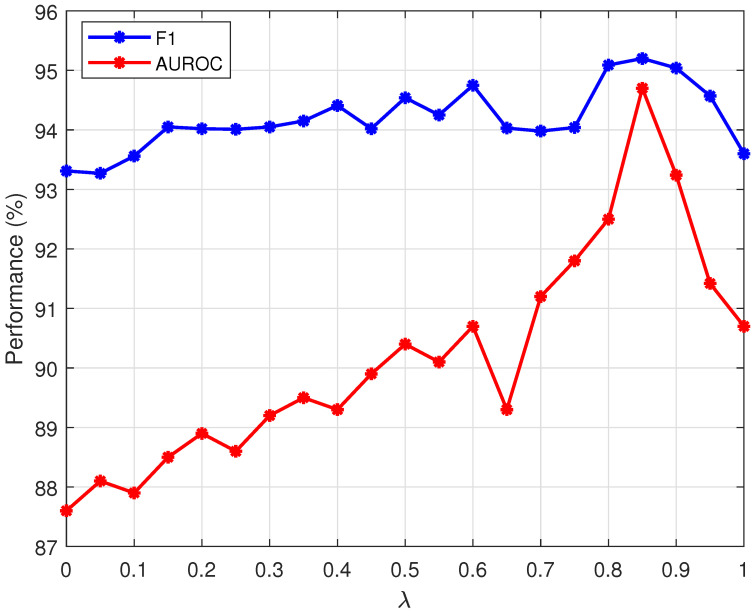
Line graph of AUROC and F1 changes.

The anomaly score measurement indicators used in this paper are weighted Euclidean distance and cosine distance, which are more suitable for the tablet defect detection task than a single indicator. In the calculation formula of the anomaly score, the weight $\omega_{i}$ of the anomaly map of different scales is obtained by network training, while the weighted values of Euclidean distance and cosine distance are obtained by the grid search method. Table 5 lists the data with λ as 0 (cosine measurement), 1 (Euclidean measurement), and 0.85 (optimal value of grid search method); Figure 11 is a trend chart of the dual indicators AUROC and F1 changing with λ, which is used to show the results of the grid search method. The best value obtained in this experiment is 0.85.

#### 4.4.4. Physical Verification

**Figure 12:**
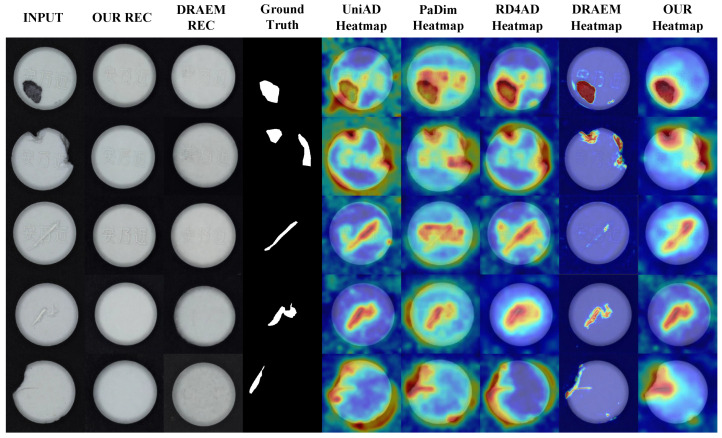
Qualitative illustration on physical verification dataset.

To verify the effectiveness of the algorithm in practical production scenarios, a complete data acquisition system was built using an MV-CS050-10UC camera, various LED light sources, and an XCY-ICS400-V2 line scan test rig platform. This system was used to construct a comprehensive tablet defect detection dataset that includes samples of normal, defective, contaminated, and scratched tablets. This dataset is designed in the MVTec AD format, and the dataset contains 1000 normal tablets and more than 1500 abnormal defect photos. For this dataset, the algorithm in this paper is compared with the four algorithms of UniAD, PaDiM, RD4AD, and DRAEM to fully verify the performance of this method in the task of tablet defect detection. The experimental results are shown in Table 6, and the visualization results are shown in Figure 12.

As shown in Table 6, in the field validation dataset, the proposed algorithm is still superior to the other four algorithms in various indicators, and compared with the best data, AP, AUROC, F1, and PRO improved by 1.54%, 3.26%, 5.60%, and 2.7 8%, respectively. It can also be seen from Figure 12 that when faced with interference factors such as complex tablet textures and diverse defect shapes, DRAEM missed detections, oversimplified abnormal expressions of abnormal areas, and had weak overall detection robustness. UniAD and other algorithms had different degrees of insufficient thermal differentiation between the background and defects and were prone to misjudgment in complex defect scenes. In contrast, the background area of the proposed method was thermally clean; the abnormal area was thermally focused, had clear boundaries, and had better abnormality detection effects.

## 5. Conclusions

In order to solve the problems of diversified defects, few defect samples, the poor reconstruction effect of unsupervised methods based on reconstruction, and the inaccurate positioning of abnormal areas in the tablet production process, this paper proposes an unsupervised tablet defect detection method (DTDD) based on a diffusion model. We propose an Assisted Reconstruction (AR) network and Semantic-Enhanced Lightweight Attention to help the diffusion model achieve better reconstruction results, and we use the Scale Fusion (SF) network in the feature matching part to improve the matching accuracy. Finally, this paper designs anomaly scores and visualization heat maps for tablet defect detection tasks to comprehensively and reasonably evaluate and display the anomaly detection effect. A large number of experimental results on tablet defect datasets show that the algorithm in this paper is superior to advanced methods in the same field, and both positioning and detection performance are greatly improved. In the future, we will use more data to train the model and expand the training target to more types of pharmaceutical products so that it can play a greater role in the pharmaceutical testing industry.

## Figures and Tables

**Table 1:** Comparative experiment with baseline model.

Algorithmic	AP (%)	F1 (%)	AUROC (%)	PRO (%)
LDM	86.27 ± 3.75	85.62 ± 3.38	80.13 ± 2.42	75.91 ± 5.93
Ours	97.42 ± 1.47	95.23 ± 1.34	94.74 ± 1.02	91.64 ± 1.53

**Table 2:** Quantitative results on Tablet dataset.

Algorithmic	AP (%)	F1 (%)	AUROC (%)	PRO (%)
UniAD	93.52 ± 3.02	92.07 ± 2.43	89.14 ± 1.29	87.73 ± 3.58
PaDiM	91.41 ± 1.76	90.82 ± 1.38	87.55 ± 0.79	84.91 ± 2.47
RD4AD	94.57 ± 1.54	92.68 ± 1.97	90.43 ± 1.06	89.28 ± 2.78
DRAEM	90.86 ± 3.32	88.32 ± 3.01	86.19 ± 1.65	83.36 ± 4.76
YOLOV8	96.72 ± 4.35	96.01 ± 4.03	91.31 ± 2.87	84.62 ± 6.32
Ours	97.42 ± 1.47	95.23 ± 1.34	94.74 ± 1.02	91.64 ± 1.53

**Table 3:** Ablation studies of SELA.

Algorithmic	AP (%)	F1 (%)	AUROC (%)	PRO (%)
Self-Attention	90.38 ± 1.80	89.76 ± 1.75	85.52 ± 1.62	85.07 ± 1.85
CBAM	94.92 ± 1.53	93.22 ± 1.45	90.78 ± 1.35	89.63 ± 1.67
ECA-Net	95.28 ± 1.42	95.73 ± 1.31	89.89 ± 1.28	88.88 ± 1.59
Ours	97.42 ± 1.47	95.23 ± 1.34	94.74 ± 1.02	91.64 ± 1.53

**Table 4:** Ablation studies of SFN.

Algorithmic	AP (%)	F1 (%)	AUROC (%)	PRO (%)
ResNet50	94.38 ± 1.52	92.22 ± 1.41	92.04 ± 1.33	88.41 ± 1.25
ResNet101	93.57 ± 1.63	92.35 ± 1.32	91.32 ± 1.42	87.19 ± 1.34
VGG16	90.68 ± 1.14	89.48 ± 1.23	89.25 ± 1.15	82.75 ± 1.08
VGG19	91.07 ± 1.35	89.81 ± 1.44	87.97 ± 1.22	83.41 ± 1.16
Efficient net B0	93.43 ± 1.46	90.27 ± 1.35	89.83 ± 1.34	85.68 ± 1.27
Efficient net B4	93.92 ± 1.55	90.53 ± 1.43	90.46 ± 1.26	86.14 ± 1.32
Inception net V3	91.98 ± 1.24	91.46 ± 1.33	91.11 ± 1.25	85.97 ± 1.17
Inception net V4	92.67 ± 1.33	91.40 ± 1.42	90.88 ± 1.34	86.52 ± 1.24
ResNet+FPN	95.14 ± 1.78	92.89 ± 1.53	92.64 ± 1.56	89.31 ± 2.06
Ours	97.42 ± 1.47	95.23 ± 1.34	94.74 ± 1.02	91.64 ± 1.53

**Table 5:** Ablation studies of anomaly score.

λ	AP (%)	F1 (%)	AUROC (%)	PRO (%)
0	94.31 ± 1.43	93.34 ± 1.42	87.58 ± 1.23	89.37 ± 1.62
0.85	97.42 ± 1.47	95.23 ± 1.34	94.74 ± 1.02	91.64 ± 1.53
1	96.23 ± 1.49	93.58 ± 1.39	90.74 ± 1.15	89.73 ± 1.71

**Table 6:** Quantitative comparisons on physical verification dataset.

Algorithmic	AP (%)	F1 (%)	AUROC (%)	PRO (%)
UniAD	91.68 ± 3.21	90.55 ± 2.59	88.08 ± 1.45	85.52 ± 3.71
PaDiM	89.27 ± 1.93	88.74 ± 1.55	85.73 ± 0.96	82.89 ± 2.63
RD4AD	94.89 ± 1.71	91.01 ± 2.13	88.21 ± 1.23	87.27 ± 2.93
DRAEM	88.51 ± 3.55	86.29 ± 4.19	83.96 ± 1.81	80.44 ± 4.91
Ours	96.43 ± 1.64	94.27 ± 1.53	93.81 ± 1.11	90.05 ± 1.68

## Data Availability

Dataset available on request from the authors.
